# Autoimmune T-Cell Reactivity to Myelin Proteolipids and Glycolipids in Multiple Sclerosis

**DOI:** 10.1155/2013/151427

**Published:** 2013-11-07

**Authors:** Judith M. Greer

**Affiliations:** UQ Centre for Clinical Research, Royal Brisbane & Women's Hospital, The University of Queensland, Brisbane, QLD 4029, Australia

## Abstract

Central nervous system (CNS) myelin, the likely major target of autoimmune attack in multiple sclerosis (MS), contains a number of unique components that are potential targets of the attack. Two classes of molecules that are greatly enriched in CNS myelin compared to other parts of the body are certain types of proteolipids and glycolipids. Due to the hydrophobic nature of both of these classes of molecules, they present challenges for use in immunological assays and have therefore been somewhat neglected in studies of T-cell reactivity in MS compared to more soluble molecules such as the myelin basic proteins and the extracellular domain of myelin oligodendrocyte glycoprotein. This review firstly looks at the makeup of CNS myelin, with an emphasis on proteolipids and glycolipids. Next, a retrospective of what is known of T-cell reactivity directed against proteolipids and glycolipids in patients with MS is presented, and the implications of the findings are discussed. Finally, this review considers the question of what would be required to prove a definite role for autoreactivity against proteolipids and glycolipids in the pathogenesis of MS.

## 1. Central Nervous System Myelin

**Figure 1:**
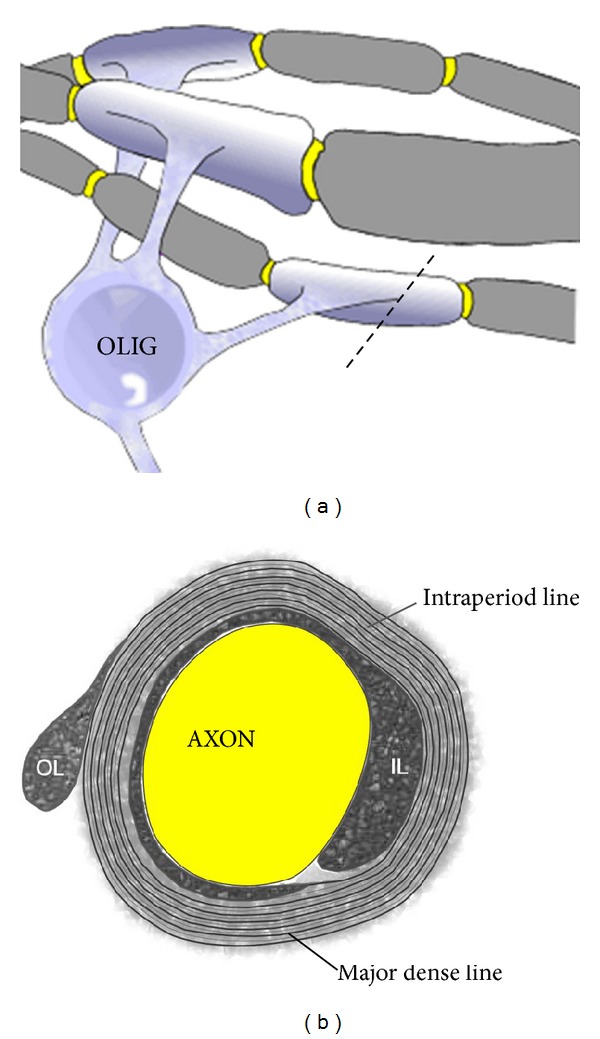
Stylized diagram of myelinated nerve fibres in the CNS. (a) Myelin is produced by the oligodendrocyte (OLIG) and is laid down in segments along nerve axons. Single oligodendrocytes can myelinate up to 50 segments. The area of bare axon between each myelinated segment (shown in yellow) is called the node of Ranvier. Electrical impulses move down the axon by “jumping” from one node to another in a process known as saltatory conduction. A cross-section through the myelinated segment at the dotted line is shown in (b). (b) The myelin sheath forms from flattened cytoplasmic processes from the oligodendrocyte that are elaborated around the axon and which then compact their cytoplasmic content (except for small pockets at the periphery of the membrane, which can appear as the inner loop (IL) immediately adjacent to the axon and the outer loop (OL) at the outer edge of the membrane). The major dense line represents the compacted cytoplasm. The intraperiod line is formed by close apposition of the membrane layers.

In multiple sclerosis (MS), damage selectively affects the central nervous system (CNS). Irrespective of the event(s) that initiate the damage to the CNS in MS, most studies agree that development of autoreactivity against molecules in the CNS myelin sheath is the most likely mechanism underlying the chronic relapsing and progressive nature of MS. Myelin is a unique lipid-rich specialized membrane found only in the nervous system of vertebrates. It acts as an insulator of the nerve axons and allows nerve impulses to pass rapidly along the axon in a series of jumps (saltatory conduction) from one gap between myelinated segments (node of Ranvier) to another, and also provides trophic support to the axon and maintains the integrity of the axon. Myelin is present in both the CNS, where it is produced by oligodendrocytes, and the peripheral nervous system (PNS), where it is produced by Schwann cells; however, there are distinct differences in the makeup of the myelin in the CNS and PNS (Table 1), and only the CNS myelin is damaged in MS. In addition, Schwann cells only myelinate a single segment of an axon, whereas each individual oligodendrocyte can myelinate numerous separate segments of axons in the CNS (Figure 1(a)). The oligodendrocytes extend membrane sheets which wrap around segments of axon and compact to form the multilamellae structure of compact myelin (Figure 1(b)). Despite the importance of the compact lamellar structure of myelin in enhancing nerve transmission, the molecular basis of this complex structure is still unclear.

A number of the lipids and proteins that make up CNS myelin are either found exclusively in the CNS or are found at higher concentrations in the CNS than in other parts of the body, and targeting of these molecules is likely to explain why autoimmune cells enter and cause damage just to the CNS in MS. Within compact CNS myelin, the distribution of the myelin proteins throughout the myelin lamellae varies from protein to protein, whereas the distribution of lipids is more uniform [1].

### 1.1. Proteins of CNS Myelin

#### 1.1.1. Myelin Proteolipids

Proteolipids are ubiquitous integral membrane lipoproteins that are soluble in organic solvents and insoluble in water. They occur as membrane components in many plant, animal, and bacterial cells but are most abundant in CNS white matter. In CNS myelin, myelin proteolipid protein (PLP) and its alternatively spliced isoform, DM20, which is identical to PLP, apart from deletion of a 35 amino acid intracellular segment (PLP residues 116–150), make up the bulk of the proteolipids [2]. PLP is the more abundant isoform in CNS myelin. Together, PLP and DM20 constitute >50% of the total protein of CNS myelin. PLP is very highly conserved, with human and rodent PLP being identical. *PLP1*, the gene encoding PLP and DM20, is located on the X chromosome; multiple copies of *PLP1*, deletion of *PLP1*,or point mutations in *PLP1* can lead to the X-linked dysmyelinating disorders, Pelizaeus-Merzbacher disease (PMD), and spastic paraplegia type 2 (SPG2) [3, 4].

**Figure 2:**
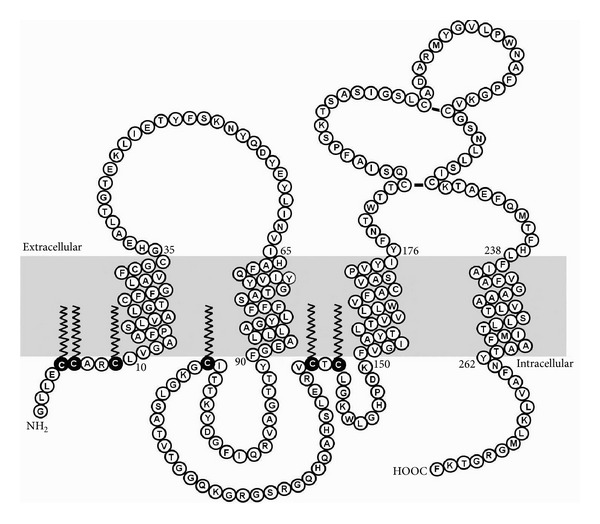
Topology of PLP in the membrane (shaded area). Amino acids are indicated using the single letter code. Cysteine residues that are thioacylated *in vivo* are shown as black circles with C printed in white, with the lipid tail shown as a zigzag line.

PLP is a highly hydrophobic protein, and its hydrophobic character is increased further by posttranslational modification of the protein in the form of covalent attachment of long-chain fatty acids (Figure 2). Approximately 3-4% by weight of PLP is made up of fatty acids, predominantly palmitic acid (60%), with lesser amounts of oleic and stearic acids, occurring in thioester linkage with cysteines at positions 5, 6, 9, 108, 138, and 140 of the protein [5, 6].

PLP has affinity for phospholipids and cholesterol and is thought to play a major role in the stabilization of compact myelin by effecting membrane cohesion at the extracellular side, that is, at the intraperiod line [7]. In patients with PMD or SPG2, the myelin that forms is poorly compacted. However, PMD patients who have *PLP1* mutations that cause greatly reduced amounts of PLP, but not DM20, or mutant mice expressing DM20, but not PLP, show a slow disintegration of the compact layers of the myelin sheath with aging, which also suggests a role for the intracellular loop of PLP in stabilizing compact myelin [8–10]. It has also been suggested that PLP interacts with integrin receptors to affect signal transduction in oligodendrocytes and homing of immature oligodendrocytes [11].

Although oligodendrocytes are the predominant cell types that express PLP/DM20, recent work has shown that, at least in rodents, PLP is also expressed by a subgroup of neurons in the brainstem [12]; the role of this neuronal PLP expression is not currently known. In addition, very small amounts of PLP/DM20 (~0.05% of total protein) are produced by Schwann cells in the PNS, with DM20 being the dominant isoform, but they are not inserted into the PNS myelin membrane [13]. Interestingly, however, the absence of PLP, but not DM20, in Schwann cells can lead to the development of peripheral neuropathy, suggesting that the 35 amino acid PLP-specific domain plays an important role in normal peripheral nerve function [14].

It is thought that PLP and DM20 are likely major targets of the autoimmune response in MS [15], since they are highly encephalitogenic in animals [16–20], and autoreactivity directed against PLP is elevated in patients with MS, as discussed further below.

Two other proteolipids found in CNS myelin are plasmolipin [21, 22] and MAL/VIP17 [23, 24], which share some sequence homology. These are minor components of CNS myelin and are also found in PNS myelin and in many other tissues, making them less likely candidates as target antigens in MS. There have not been any studies to determine if these proteolipids are a target of autoimmune responses in MS.

#### 1.1.2. Other Myelin Proteins

After PLP/DM20, the next most abundant group of CNS myelin proteins is the myelin basic proteins (MBP). There are several isoforms of MBP, which together make up about 30% of the total myelin protein in both the CNS and the PNS. MBP has a highly basic isoelectric point and is believed to act as “glue” between the cytoplasmic faces of the myelin membrane leaflets through electrostatic interactions with negatively charged phospholipids. MBP is also thought to play multiple essential roles in morphogenesis of the myelin-producing cells and stabilization of actin fibres in compact myelin [25]. Numerous studies have investigated autoreactivity directed against MBP in MS [26–43], and several MBP-specific therapeutic approaches have been tested in MS patients, with limited success [44–47].

Oligodendrocyte-specific protein (OSP or claudin 11), an integral membrane protein which makes up around 7% of the total CNS myelin protein [48], is thought to play a role in the formation of tight junctions within myelin sheaths and to be involved in regulating proliferation and migration of oligodendrocytes [49]. OSP is also thought to play a role, together with PLP, in the maintenance of normal compact myelin [50], although the mechanism by which this occurs is still not well understood. OSP also adds to the electrical resistance of myelin by preventing leakage of charged ions and electrical current through the spiral space between myelin layers [51]. OSP is encephalitogenic in mice [52] and elevated T-cell responses to OSP have been reported in a small proportion of patients with MS in two studies [53, 54].

Less abundant myelin proteins include myelin oligodendrocyte glycoprotein (MOG), the myelin-associated glycoprotein (MAG), and the enzyme 2′,3′-cyclic nucleotide 3′-phosphodiesterase (CNPase). MOG has an unusual distribution, being found only on the outermost turn of the myelin membrane; its function remains unclear, but it is possible that it has a role in transducing signals through the membrane to the cytoskeleton [55]. MOG has been the subject of intense research in MS and the experimental autoimmune encephalomyelitis (EAE) model of MS over the last 10 years or so, and there are numerous publications on the topic of autoimmune reactivity to MOG protein and peptides [26, 29–31, 56–65].

MAG is found on the periaxonal loop and paranodal loops of myelin and functions both as a ligand for an axonal receptor (a sialylated glycoconjugate, probably a ganglioside [66, 67]), that is needed for the maintenance of myelinated axons, and as a receptor for an axonal signal that promotes the differentiation, maintenance, and survival of oligodendrocytes [68]. There have been a small number of reports of elevated T-cell responses to MAG in MS patients [69, 70], as well as in noninflammatory polyneuropathy.

CNPase is a lipid-anchored membrane-surface component that is confined to oligodendrocytes and to noncompact cytoplasm-containing regions of the myelin sheath [71] and which plays a role in branching and process formation of oligodendrocytes [72]. Only a small number of studies have investigated CNPase as a potential target of autoimmune attack in MS [73].

### 1.2. Myelin Lipids

**Figure 3:**
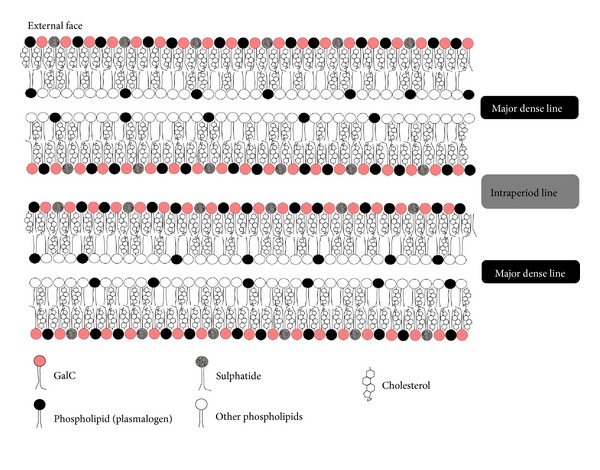
The distribution of lipids in CNS compact myelin. GalC and sulphatide are asymmetrically distributed, being highly enriched on the outer surfaces of the membranes. The molar ratio of each of the major classes of lipids is approximately as shown in the diagram.

Despite the important role that the myelin proteins play in CNS myelin, they are not the most abundant molecules in the myelin membrane; 70–85% of the total dry weight of the myelin membrane is lipid, which is a substantially higher percentage than in other membranes (which typically contain 30–50% lipid). The most abundant lipids in CNS myelin are cholesterol, phospholipids (including phosphatidylcholine, phosphatidylethanolamine, phosphatidylserine, phosphatidylinositol, and sphingomyelin), and glycolipids (predominantly galactocerebroside (GalC) and sulphatide, with lesser amounts of gangliosides), in a molar ratio of around 2 : 2 : 1. The lipids are present in an asymmetrical arrangement, with the glycolipids clustering on the external side of the myelin membrane (Figure 3) [1, 74]. Since lipids make up such a large proportion of the myelin membrane, their possible role in the pathogenesis of MS should not be overlooked. Several changes to myelin lipids have been reported in MS, including decreases in lipid content in normal appearing white matter [75, 76], increased oxidized phosphatidylcholine levels [77], and a shift in the lipid composition to a higher phospholipid and lower sphingolipid content [75].

**Figure 4:**
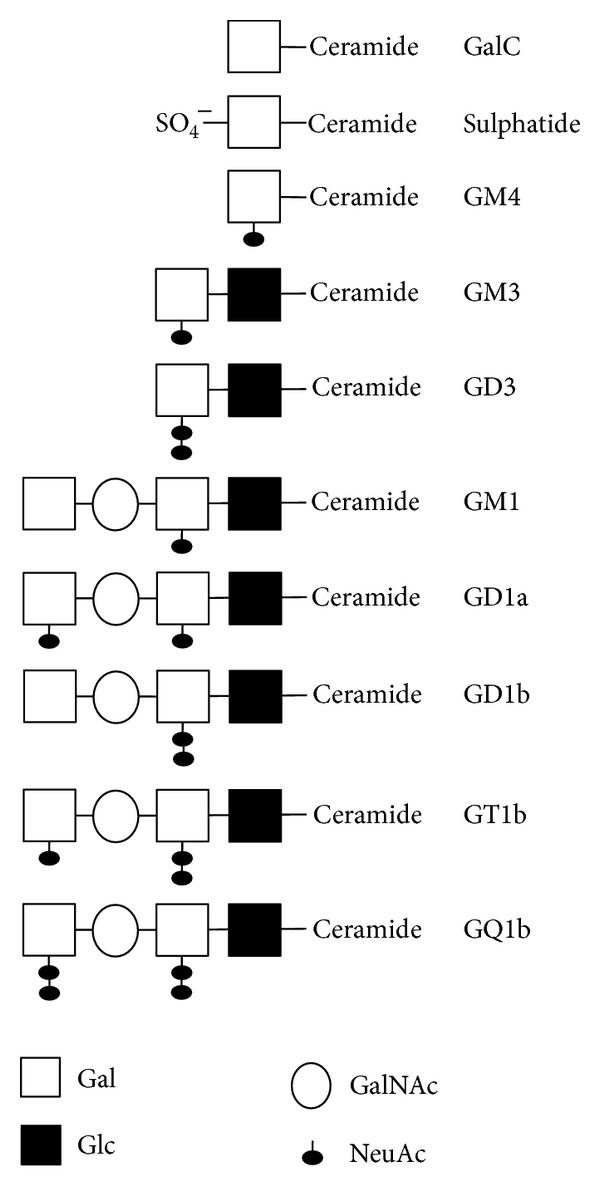
Schematic showing the relationship between the galactosylceramides (GalC and sulphatide) and some of the gangliosides present in the CNS [84, 176]. The nomenclature of the gangliosides indicates whether they are mono-, di-, tri-, or tetrasialogangliosides (GM, GD, GT, and GQ, resp.), their order of migration on thin-layer chromatograph (e.g., GM3 migrates further than GM1), and variations in basic structure (e.g., GD1a versus GD1b). GalC: galactocerebroside; Glc: glucose; Gal: galactose; GalNAc: N-acetylgalactosamine; NeuAc: N-acetylneuraminic acid (sialic acid).

The cholesterol and phospholipid content of CNS myelin resembles that of other plasma membranes and makes it somewhat unlikely that they are major targets of autoimmunity in MS. In contrast, the glycolipids that occur in greatest abundance in CNS myelin are expressed to a lesser extent in other tissues. The myelin glycolipids all contain ceramide as a basic subunit and can be subdivided into cerebrosides and gangliosides, depending on the attachment of different oligosaccharide groups to a sphingoid base (Figure 4).

The two cerebrosides most highly enriched on the surface of the oligodendrocyte cell body and throughout the myelin membrane, compared to other parts of the body, are the galactosylceramides, GalC and sulphatide, which together make up 25–30% of human CNS lipids. Most other membranes in the body have a higher concentration of glucosylceramides than galactosylceramides [1]. Studies of mice lacking an enzyme critical for synthesis of GalC and sulphatide show marked conduction abnormalities [78], suggesting that these lipid components are essential for the insulative capacity of the myelin sheath. It appears that GalC and sulphatide may also play overlapping roles with PLP in stabilizing the intraperiod line of compact myelin [79]. GalC and sulphatide are also essential for the proper formation of the CNS node of Ranvier [80, 81], and mice lacking these molecules exhibit disorganized paranodal structures and progressive dysmyelination in the CNS, although PNS myelin is structurally unaffected [82]. Intriguingly, the composition/distribution of some low-abundance myelin proteins that regulate functions such as cytoskeletal dynamics, energy metabolism, vesicular trafficking, or adhesion is also changed in these mice, supporting a role of these lipids in intracellular trafficking [83]. In addition, antibody-mediated signalling through GalC leads to rearrangement of microtubules in oligodendrocytes [55]; since GalC first appears on the surface of the cell at the stage of transition from a progenitor cell to an immature oligodendrocyte, it has therefore been suggested that GalC may transduce signals critical for oligodendrocyte development. 

Gangliosides are another class of glycosphingolipids that contain one or more sialic acid residues attached to their oligosaccharide chain. Gangliosides are predominantly enriched in the plasma membranes of neurons and axons; however, they are also minor components of myelin (~0.1–0.3% w/w), with GM3, GM4, and GD3 occurring most commonly in myelin [84]. GM4 is generated from GalC by addition of sialic acid, whereas the biosynthesis of the other gangliosides involves conversion of glucosylceramides to lactosylceramide and the subsequent addition of sialic acid moieties.

Gangliosides on axons interact with MAG in myelin to facilitate adherence of the first loop of myelin around the nerve axon [66, 67]; however, the role of the myelin gangliosides is not clear. It has been suggested that they might bind to growth factor tyrosine kinase receptors, thus regulating their activity [85], and it has been shown that addition of GM3, but not other mono- or disialylated ganglioside species, enhances the differentiation of oligodendrocytes in culture [86].

## 2. T-Cell Responses to Myelin Proteolipids and Glycolipids in MS

In the sections below, what is known of T-cell autoreactivity directed against two of the abundant CNS myelin components, namely, the proteolipids and glycolipids, will be reviewed. Autoimmune reactivity targeting CNS myelin components is thought to be a major mechanism for the development of MS; however, particularly when considering T-cell autoreactivity, it is important to remember that not all MS patients will necessarily show elevated levels of autoimmune responses to myelin antigens at any single time point tested, as, unlike antibodies, which can persist for many months, T-cell responses are typically quite transient. In addition, because the clinical and neuropathological features of MS change markedly with time, it is therefore likely that autoimmune T-cell responses might change with time from onset of disease, as well as during fluctuations of disease activity. Whereas some studies of autoreactivity against myelin antigens have grouped patients according to their disease course and/or activity, others have not. In addition, some of the studies have used T cells immediately after they have been isolated from blood or cerebrospinal fluid (CSF) of MS patients, whereas others have used various nonspecific *in vitro* activation methods to enhance the numbers of myelin-specific cells prior to assaying for autoreactivity. Thus, there is a great deal of variability in experimental systems, which may lead to variability in results.

Another variable in assays of T-cell reactivity to myelin antigens, particularly when dealing with myelin proteins extracted from brain tissue, is the species of the donor tissue. Human tissue has been used in some experiments, but often bovine or rodent myelin has been used as a tissue source, and it is important to note that the sequences of the myelin proteins from these different tissues are not always identical and that the relative proportions of myelin lipids vary slightly from one species to another. In addition, the antigen concentration used to stimulate the T cells *in vitro* has been shown to play a role in the outcome of the response [87], and while some studies have investigated a wide range of antigen concentrations, others report results from only a single antigen concentration.

### 2.1. T-Cell Autoimmunity Directed against PLP/DM20

#### 2.1.1. T-Cell Reactivity to Whole PLP Preparations

Immune reactivity to whole PLP in patients with MS has been investigated by various groups worldwide [59, 88–98]. Working with whole PLP preparations presents several challenges, from the point of view of immunological assays, because of the hydrophobicity of PLP and its solubility only in chloroform : methanol, which is not compatible with most immunological assays. The PLP apoprotein can be converted to a “water soluble” form; however, it is not stable and PLP easily precipitates out of solution, particularly in the presence of salts (e.g., in tissue culture medium). The ease with which the PLP precipitates out of solution depends particularly on the degree of delipidation achieved; the more lipid remaining, the greater the chance that it will precipitate out. But even PLP preparations containing little lipid can precipitate out of solution, forming a flocculent mass that floats in the tissue culture vessel. Cells either become suspended in this mass and cannot come into contact with each other or sink to the bottom of the vessel, below the PLP. Neither of these conditions favours T-cell activation.

In addition, some of these studies used bovine PLP as the antigen. One Swedish group did successfully use bovine PLP to show enhanced levels of autoreactivity in blood and CSF of MS patients [89, 90, 95]; however, most studies using bovine PLP were not particularly successful in demonstrating an increased frequency of autoreactive PLP-specific T cells in MS patients [32, 59, 88, 96]. Bovine PLP has >98% homology with human PLP; however, there are amino acid differences at three of the 276 residues, at positions 88, 188, and 198. Recent studies have shown that some of the immunodominant epitopes of PLP for humans fall within the 180–230 region of PLP [53, 92, 98–100]. Thus, bovine PLP may not be able to elicit strong immune responses by T cells that are specific for the human 180–230 region, given that 2 of the amino acid differences between human and bovine PLP lie within this region. Some HLA types may be able to successfully present these epitopes, but in others, the amino acid differences may render the peptide nonimmunogenic. In mice, which have exactly the same PLP sequence as humans, changing the residue at 188 to that of the bovine sequence abolishes the encephalitogenicity of PLP peptide 178–191 and changes the recognition pattern by T cells [101].

Studies utilizing human PLP have been more uniform in their ability to detect increased responsiveness in blood or CSF from MS patients compared to healthy controls or patients with other neurological diseases. Trotter et al. [91] found, in a small series of patients, that patients with a rapidly progress course of MS showed increased reactivity to PLP, compared to MS patients in remission and healthy controls. Subsequently, further studies from the same group [93, 94, 98] and others [92] showed that, while human PLP induced only moderate reactivity by T cells from most MS patients, T-cell lines generated by stimulation with human PLP were highly reactive against specific PLP peptides (see below). In a longitudinal study of 7 MS patients, Hellings et al. [97] found fluctuating frequencies of IFN-*γ* secreting T cells after stimulation with human PLP or other myelin antigens in ELISPOT assays. In several of these patients, substantial increases in the frequency of PLP-specific T cells occurred in conjunction with new clinical and/or MRI activity.

#### 2.1.2. T-Cell Responses to PLP Peptides

Because of the difficulties encountered when using whole PLP in immune assays, most studies of PLP have favoured the use of PLP peptides [26, 28, 53, 87, 91–94, 97–100, 102–125]. Even so, many of the peptides of PLP are very hydrophobic and poorly soluble and can still precipitate out of solution in the presence of salts; slight acidification of the peptides helps prevent some of these problems [53, 92].

Some of the first studies testing T-cell reactivity to PLP peptides in humans [28, 91, 108, 112] used two peptides that had been identified as encephalitogenic epitopes in mice, namely PLP103–116 and PLP139–151 [18, 19]. These peptides induced little reactivity in humans, and there was little difference in the frequency of T cells from MS patients or controls capable of responding to them. Subsequent studies have shown that these peptides bind only poorly to human class II HLA molecules commonly found in patients with MS, including DR2 (DRB1*15 : 01; DRB5*01 : 01), DR3, and DR4 [99], which likely explains this lack of responsiveness, although a recent study using HLA transgenic mice suggests that PLP139–151 can be presented by DQB1*06 : 02 [126]. One of the most important determinants of whether individuals can respond to a peptide is whether they carry the appropriate HLA molecules to allow presentation of that peptide to T cells. In some of the studies outlined in this section, details are given of HLA types of patients and controls, but most do not have this information, making interpretation of results somewhat difficult. HLA DRB1*15 : 01 is known to be associated with MS, particularly in Caucasian populations, but even then it is only present in 60–65% of patients, and most patients are not homozygous for DRB1*15 : 01, which means that there are a large number of other HLA class II molecules, in addition to HLA class I molecules, that could be important for presentation of protein antigens in MS. 

Subsequent studies tested a broader range of peptide, although the focus was still on the first intracellular (and relatively hydrophilic) loop of PLP. It was shown that many T-cell lines established by their reactivity to whole PLP showed specificity for PLP40–60 and PLP89–106, both in their ability to proliferate in response to peptide and to lyse PLP peptide-pulsed autologous targets [93, 94]. The T cells responding to these peptides were predominantly CD4^+^ MHC class II-restricted (generally HLA-DR4 restricted for PLP40–60 and HLA-DR2 restricted for PLP89–106).

Using shorter peptides from these same regions of PLP, it was also shown that CD8^+^ PLP-specific cytotoxic T cells (CTL) could be generated from MS patients [103], with PLP45–53 being identified as an HLA-A3 epitope and PLP80–88 being presented by HLA-A2. These CTL could secrete proinflammatory cytokines, chemokines, and matrix metalloproteinases [103] and were cross-reactive with a peptide from the yeast *Saccharomyces cerevisiae*, suggesting that organisms commonly encountered in the environment may be able to induce CTL that are cross-reactive with myelin antigens [113]. A later study, which used 2 large peptide pools of PLP peptides to stimulate CD4^+^ and CD8^+^ T-cell responses, also reported that the frequency of CD8^+^ PLP-specific T cells was significantly increased in MS patients compared to controls [109].

Meanwhile, other studies using overlapping peptides covering the whole of the human PLP sequence also identified peptides from other regions of PLP that induced increased reactivity in T-cell lines generated by multiple stimulations with whole human PLP [99], or by T cells isolated directly from MS patients [92] and patients with first demyelinating events suggestive of MS [115]. These showed an additional immunodominant region of PLP in the second extracellular loop (residues 180–230), which had also been shown to contain a cluster of encephalitogenic epitopes for multiple mouse strains [17]. Reactivity to this region of PLP is increased particularly in patients who carry specific alleles of DR4, DR7, or DR13 [53, 92].

An increased frequency of T cells responding to the PLP30–60 and PLP180–210 regions was confirmed in several additional studies using PLP-reactive T-cell lines [98, 117, 123] or *ex vivo* testing of cells obtained directly from blood [53, 100]. These PLP-specific T cells, when isolated from relapsing-remitting MS patients during an exacerbation, were found to produce substantially more of the proinflammatory cytokines IFN-*γ* and TNF-*α* than did T-cell lines derived from patients in remission or from healthy controls [117, 123]. In addition, enumeration of IFN-*γ*-secreting T cells directly from blood of MS patients and controls demonstrated a highly increased frequency of PLP-peptide-reactive T cells in MS patients versus controls [119].

Sexual dimorphism in the T-cell response to PLP peptides has also been noted, with females (both MS patients and healthy controls) typically showing increased responses to PLP, but not to MBP, compared to males [63, 120, 121]. The data suggest a gender bias towards PLP-specific Th1 responses in females with MS, and an overall elevated level of reactivity to PLP peptides in females, both of which may contribute to the female predominance in MS. There are several potential explanations for the differences in reactivity to PLP in males and females. Firstly, it is known that females show more robust responses to a variety of antigens than do men, largely due to the effects of gonadal hormones [127], and one study has shown that estrogens selectively modify cytokine secretion in PLP-specific CD4^+^ T-cell clones isolated from patients with MS, and from normal control subjects [104]. Alternatively, the explanation may relate to the populations of patients tested in these studies, as it has previously been suggested that patients with primary progressive (PP-MS) are less likely to show elevated T-cell reactivity to PLP than patients with relapsing-remitting MS (RR-MS) [92]. Since the ratio of males to females in the subgroup of patients with PP-MS is much higher (1 : 1) than that in the RR-MS subgroup (1 : 3), an excess of males with a PP-MS disease course could skew the proportions of male and female responders in these studies. Another possible explanation for a gender effect in T-cell responsiveness to PLP may be related to the presence of the gene encoding PLP on the X chromosome and/or to differential effects of sex hormones on expression of DM20 in thymic epithelial cells, thereby modulating induction of tolerance to PLP. Irrespective of the underlying reasons for the observations of sexual dimorphism in the response to PLP, given that females show an increased incidence of MS compared to males and that most female patients show remission from MS during pregnancy, there is a very strong case that all MS T-cell data should be analysed on the basis of gender.

#### 2.1.3. Role of Fatty Acids in T-Cell Responses to PLP

As noted earlier, PLP is posttranslationally thioacylated by attachment of fatty acids at up to 6 cysteine residues within the protein. Thiopalmitoylated PLP peptides PLP103–116(Cys108-Palm) and PLP139–151(Cys140-Palm), which occur naturally in intact PLP, were shown to be significantly more immunogenic and encephalitogenic in mice than the corresponding nonpalmitoylated peptides [128]. The thiopalmitoylated peptides were taken up much more rapidly and effectively than the non-palmitoylated peptides into endosomes (and hence the MHC class II presentation pathway) within antigen-presenting cells (APC) [129]. This rapid concentration of peptide in the endosomes was facilitated through the presence of the palmitic acid, as fatty acids containing ≥14 carbons can passively diffuse across cellular membranes, carrying attached peptides with them. In addition, the thioester bond between protein and fatty acid was easily broken down by thioesterases in APCs, thus stranding the peptide portion of the molecule in the endosome [129]. It is not yet known whether naturally thioacylated peptides of PLP that are released following myelin damage in MS might induce enhanced reactivity by T cells from MS patients. It is of interest to note, however, that thioacylation of PLP is increased threefold in spontaneously demyelinating transgenic mice, compared to wild-type mice, and it has been speculated that increased thioacylation of PLP also occurs in MS [130].

#### 2.1.4. Are T Cells Specific for PLP Pathogenic?

Currently, it is not possible to prove conclusively that T cells specific for PLP or any other myelin antigen are definitely pathogenic in patients with MS, or whether they are an epiphenomenon, produced secondary to the release of myelin by the demyelinating process in MS. Several approaches have been taken to try to address this question. One approach has been to assess the potential functional effects of autoreactive T cells, by determining whether or not they produce proinflammatory cytokines, chemokines, or other effector molecules that appear to be essential for encephalitogenicity of T cells in animal models of MS. Thus, a Th1 and/or Th17 phenotype is typically considered to be pathogenic, whereas a Th2 phenotype is considered to be indicative of a protective T cell. As noted above, several studies have shown that both CD4^+^ and CD8^+^ T cells from MS patients typically make a Th1-skewed response to PLP peptides. At present, there have not been any reports from human studies as to whether or not some PLP-specific T cells are of a Th17 phenotype, although studies on experimental animals would suggest that this will be the case [52, 126]. It has also been reported that the cytokine secretion profile of PLP-specific T cells, but not that of tetanus toxoid-specific T cells, changes from a predominantly Th1 to predominantly Th2 type upon treatment with cyclophosphamide plus methylprednisolone [131].

Another approach to assessing the potential pathogenicity of PLP-specific T cells has been to determine if changes in frequency of the cells correlate with MRI or clinical evidence of disease activity in patients with RR-MS. Two longitudinal studies, one using limiting dilution analysis of the frequency of PLP41–58- and PLP184–209-reactive T cells over an 18 month period in 5 relapsing-remitting MS patients and 4 healthy controls [100] and the other testing the response to whole human PLP in 7 MS patients and 2 healthy controls by IFN-*γ* ELISPOT assays [97], have been reported. In both studies, the overall frequency of PLP-specific T cells was higher in MS patients than in healthy controls. Furthermore, surges in the frequency of PLP-specific T cells occurred immediately prior to the onset of new gadolinium-enhancing MRI brain lesions and/or onset of new clinical signs in some of the patients.

Expansion and persistence of myelin antigen-specific T-cell clonotypes has also been investigated. If identical T-cell receptor (TCR) complementary determining region 3 (CDR3) sequences are present in cells collected at different time points, they are very likely to be sister clones of the same clonal origin and thus represent T cells that have been activated and expanded *in vivo* [132]. One group from Japan identified clonally expanded TCR beta CDR3 amino acid motifs of some PLP-specific T cells which were homologous to those of T cells within the MS lesions, showing that the PLP-specific T cells can infiltrate MS lesions [110]. Another study [116] reported that a PLP-specific clonotype persisted in the activated T-cell compartment of 1 of 2 patients with relapsing-remitting MS for >1 year; although the majority of clonotypes from both patients could be detected only transiently, this study did not attempt to correlate the clonotypes with disease activity. In another study using whole PLP as the antigen [97], clonally expanded T-cell clones were detected not only in 7 of 7 MS patients, but also in 2 of 2 healthy controls. No direct correlation was found between the dynamic changes in TCR repertoire and clinical measures; however, clonal expansions of T-cells seemed to be associated with the appearance of active MRI lesions in 4 of 5 patients with active MRI scans and were detected prior to exacerbation in 3 of 4 patients with a relapse.

Another approach to the question of whether or not PLP-specific T cells are pathogenic has been to correlate specific T-cell reactivity with development of lesions in particular regions of the CNS. The rationale for this is that, in animal models of MS, it is clear that different myelin autoantigens induce different clinical and histological phenotypes of disease, depending on the antigen and immunization protocol used to induce disease, and on the MHC and non-MHC genes carried by the animal. Only a small number of studies have attempted to correlate T-cell autoreactivity to PLP with the areas in which lesions form [53, 115, 118]. The earliest of these studies investigated T-cell reactivity to overlapping PLP peptides covering the whole length of PLP in 10 patients with monocentric monophasic demyelinating syndromes suggestive of a first attack of MS [115], and the second investigated T-cell reactivity to pooled PLP peptides and other antigens in 5 patients with the opticospinal form of MS and 6 patients with conventional MS [118]. As autoreactivity to PLP does not occur in all patients at all times tested, these small groups of patients did not give sufficient power to show any significant correlations between T-cell reactivity to the antigens and the site of the lesions. More recently, the correlation between reactivity to PLP and the site of lesion development has been investigated in a group of 100 MS patients [53]. This study showed a strong correlation between carriage of particular HLA molecules (DR4, DR7, or DR13), increased T-cell reactivity to PLP184–209, and development of lesions in the brainstem and cerebellum. The PLP-reactive T cells were predominantly CD4^+^, although ~10% were CD8^+^. They occurred both in patients who had brainstem lesions as the first presentation of their MS (when they were present in both blood and CSF), as well as in individuals with more advanced disease. In corroboration of this finding, the earlier small study of patients with monocentric monophasic syndromes suggestive of MS [115] had found that 2 of 3 patients with anterior brainstem syndrome showed increased reactivity to the PLP180–210 region, and two of these patients also carried DR4. This finding of a correlation between development of brainstem and/or cerebellar lesions and the PLP180–230 region is of particular interest, since C3H/HeJ mice develop lesions restricted to the brainstem and cerebellum when immunized with PLP190–209 [17, 133], suggesting that autoimmune T-cell reactivity directed against the second extracellular loop of PLP can be pathogenic.

### 2.2. Autoimmune T-Cell Reactivity Directed against Glycolipid Antigens in MS

Glycolipids have been implicated as targets of autoantibody-mediated diseases, particularly those affecting the PNS [134–136]. In addition, several studies have reported increased levels of antibodies specific for glycolipids in MS [137–148]. Studies of T-cell reactivity to myelin glycolipids have been much scarcer. 

The earliest studies to describe T-cell reactivity to gangliosides and cerebrosides in MS patients detected this reactivity using the active sheep erythrocyte (E-) rosette test. This assay was developed as an *in vitro* correlate of the delayed type hypersensitivity test [149] and had been previously used to investigate T-cell reactivity to MBP in MS [150]. The E-rosette test was based on the ability of a subset of T cells to rapidly adhere to sheep erythrocytes and on an increase in the percentage of T cells with this ability following incubation with the antigen against which the T cells had been sensitized. Subsequent studies identified CD2 and CD58 as being involved in the E-rosette phenomenon [151]. Interestingly, CD58 has recently been identified as a susceptibility gene for MS [152, 153].

In the first of the studies investigating enhancement of E-rosette formation in response to lipids by T cells from MS patients, Offner and Konat found that >95% of MS patients who were clinically stable at the time of testing responded to low (ng) doses of bovine cerebrosides or gangliosides [154]. In comparison, only very low percentages of patients with other neurological diseases or healthy controls showed enhanced E-rosette formation in response to these antigens. In a follow-up study using fractionated gangliosides of bovine, murine, or human origin, it was found that the more sialic acid groups attached to the gangliosides, the greater the ability of the ganglioside to enhance the E-rosette test, so that, for example, GQ1b was a better antigen than GM1 [155]. Subsequently, Ilyas and Davison [156] described increased reactivity to a mixed ganglioside preparation, particularly in patients with chronic progressive MS or in patients actively undergoing a relapse, but not in patients in remission, at the time of testing. 

By the late 1980s, most investigators were moving away from assays such as the E-rosette test, which gave reproducible results in the hands of some investigators, but not others. Frick [157] used an autologous cytotoxicity assay to show that purified CD8^+^ T cells from MS patients compared to controls showed increased killing of autologous tanned lymphocytes that had been coated with bovine gangliosides or cerebrosides. Again, the specific T-cell activity was increased most markedly during attacks of MS. Next, Bellamy et al. [158] found that two T-cell lines (one CD4^+^ and the other CD8^+^), derived in the presence of IL2, but absence of antigen, from cerebrospinal fluid (CSF) of patients with progressive MS, subsequently responded most strongly to gangliosides. Using the leukocyte migration inhibition assay, Beraud et al. [159] also showed that T cells from MS patients undergoing an attack (compared to T cells from patients in remission or from controls) showed increased migration inhibition when incubated in the presence of human brain gangliosides.

For the next few years, there was a paucity of studies looking at the immunogenicity of gangliosides or other myelin lipids in MS. In contrast, there were numerous studies showing that gangliosides were actually immunosuppressive. These studies were driven by investigations into the lack of responsiveness by the immune system to tumours, which typically shed gangliosides [160]. In 1996, Irani et al. [161] showed that gangliosides at high concentrations suppress immune responses, but that they are blastogenic at low concentrations. Testing of gangliosides at very low concentrations had been noted in the earliest MS studies as a crucial condition for success of the assays [154]. Recent studies have rediscovered the phenomenon that large (*μ*g) amounts of myelin lipids can be immunosuppressive [162].

The 1990s saw the identification of CD1 molecules, which are nonpolymorphic MHC class I-like glycoproteins that are able to present microbial lipids and glycolipids to human T cells [163]. Shamshiev et al. showed an increased frequency (compared to controls) of circulating CD8^+^ *αβ*TCR^+^ T cells that secreted IFN-*γ* and TNF-*α* in response to a variety of glycolipids in a small group of MS patients, and, importantly, showed that ganglioside-specific T cells were restricted by CD1b [164]. A recent study has reported specific CD1a and CD1e genotypes that appear to be associated with susceptibility to MS [165].

The most recent study of T-cell-mediated glycolipid reactivity in MS tested the ability of bovine gangliosides and sulphatide to induce proliferation in T cells from MS patients (who were also subdivided on the basis of their clinical course), healthy controls, and patients with other neurological diseases of the CNS [166]. This study found significantly increased reactivity by T cells from PP-MS patients to GM3 and GQ1b; however, the phenotype of the responding cells was not addressed.

Taken together, several reproducible findings emerge: (i) MS patients with chronic or active relapses of disease typically show increased T-cell responses to glycolipids, compared to patients in remission at the time of testing and (ii) GQ1b was shown to be a target of autoreactivity in several different studies.

## 3. What Are the Next Steps Needed in Studies of T-Cell Autoreactivity to PLP and Glycolipids?

In order to gain an improved understanding of the relevance of T cell autoreactivity to PLP and glycolipids (and other myelin antigens) to MS pathogenesis, it will be important that future studies provide, as basic information, better clinical data (including disease stage and activity and sites of lesions) together with information on gender, HLA type, and so forth. In addition, determining the phenotype of the responding cells and their HLA and/or CD1 restriction would add to the value of the studies. 

New methodologies that can discern the type of response and enumerate the frequency of responding cells should be considered. Carboxyfluorescein succinimidyl ester (CFSE) is being used more commonly to monitor lymphocyte proliferation in MS patients [53, 109], and it has great potential utility, as it can be used in conjunction with fluorescent labelling of cell surface or intracellular targets. Recently, two new methodologies of potential relevance for studies of T-cell reactivity in MS have been developed, namely, on-chip activation and detection of individual antigen-specific T cells for high throughput studies [167], and the T-cell recognition of antigen presenting cells by protein transfer (TRAP) assay [168], which has recently been shown to compare favourably to other methods in the ability to quantify myelin-specific T cells in MS patients [169].

To understand more about glycolipid-specific T-cell responses and their potential role in MS, it would be useful to establish an EAE model, induced by passive transfer of glycolipid-specific T cells. However, it may be that the pathogenic potential of glycolipid-specific T cells lies in their ability to help B-cell/antibody production, and that, as in some of the peripheral neuropathies, it is the antibody responses that are directly pathogenic.

In order to prove the disease-relevance of T-cell responses directed against PLP or glycolipids, it would also be important to attempt the establishment of improved animal models. While EAE has been invaluable in increasing our understanding of the likely autoimmune process in MS, EAE is not MS and does not prove absolutely that autoimmune reactivity against the putative autoantigens causes MS. A better way would be to transfer PLP or glycolipid-specific cells directly from a patient into humanized (HLA transgenic) mice on a severe combined immunodeficiency (scid) or RAG knockout background that had been chimerized after birth with stem cells from the same patient. These xenografted mice would then not only be useful for determining the true pathogenic potential of MS autoantigens but would also be very useful models for testing antigen-specific therapeutic agents.

## 4. Summary

Proteolipids and glycolipids are major components of CNS myelin and are largely restricted to the CNS, making them rational potential targets of autoimmune attack in MS. A relatively large body of literature spread over the last 40 years strongly suggests that these molecules are targeted in MS, and that, even though they are harder to work with than some of the other more hydrophilic myelin components, they should not be ignored in future studies of T-cell autoreactivity in MS.

## Figures and Tables

**Table 1:** Some of the constituents of myelin and their relative distribution (assembled from information in [2, 48, 170–175]).

Constituent	CNS myelin	PNS myelin
Myelin proteins (make up 20–30% w/w of myelin)		
Myelin proteolipid protein (PLP)/DM20	>50%* (PLP > DM20)	0.05% (DM20 > PLP)^†^
Myelin basic protein (MBP, also known as P_1_)	30%	5–15%^††^
Oligodendrocyte-specific protein (OSP)	7%	Not detected
2′,3′-Cyclic-nucleotide 3′-phosphodiesterase (CNPase)	2–4%	0.2%
Myelin associated glycoprotein (MAG)	1%	0.1%
Myelin oligodendrocyte glycoprotein (MOG)	0.01–0.05%	Not detected
P_0_ (glycoprotein)	Not detected	50%
P_2_	<0.01%	5–10%^††^
PMP22	<0.01%	5–10%^††^
Myelin lipids (make up 70–80% of total dry weight of myelin)		
Galactocerebroside (GalC)	23%	~10%
Sulphatide	4%	~2%
Gangliosides	0.1–0.3%	~0.1%^¶^

*Percentages represent the proportion of the molecule within the whole class of molecule; for example, >50% of the total protein fraction is PLP

^†^PLP and DM20, the alternatively spliced isoform of PLP, are present at very low concentrations in Schwann cells, but are not inserted into the PNS myelin membrane.

^††^Amounts vary depending on the peripheral nerves used as a source of tissue.

^¶^One of the most abundant gangliosides in human PNS myelin is LM1, which is not present in CNS myelin.
